# Current Views on Anthracycline Cardiotoxicity in Childhood Cancer Survivors

**DOI:** 10.1007/s00246-015-1176-7

**Published:** 2015-05-05

**Authors:** Elżbieta Sadurska

**Affiliations:** Department of Pediatric Cardiology, Medical University of Lublin, Chodźki 2, 20-093 Lublin, Poland

**Keywords:** Anthracyclines, Cardiotoxicity, Cancer survivors, Children, Heart

## Abstract

Owing to their high efficacy, anthracycline antibiotics are included in numerous chemotherapeutic regimens used—often in combination with radiation therapy and/or surgery—in treatment of solid tumours and blood malignancies, both in children and adults. However, the efficacy of modern cancer treatments, owing to which the population of cancer survivors has been on the rise in recent years, may be limited by the risk of serious complications involving multiple organs and systems, including the cardiovascular system. Being an important side effect of anthracyclines, cardiotoxicity may limit the efficacy of cancer therapies in the acute phase (i.e. during the treatment) and induce the long-term sequelae, observed years after treatment completion in childhood cancer survivors. It is very important to understand the cardiotoxicity-associated mechanisms and to determine its risk factors in order to develop and/or improve the effective countermeasures. Based on published data, the paper provides an outline of current views on anthracycline cardiotoxicity and discusses such aspects as molecular mechanisms of cardiotoxicity and its clinical manifestations as well as the new preventive strategies and diagnostic techniques used for the assessment of cardiovascular abnormalities. The widespread awareness of cancer treatment-related cardiotoxicity among the healthcare professionals may significantly improve the quality of life of the childhood cancer survivors.

## Introduction

Modern cancer therapies have brought a major breakthrough in paediatric oncology within the last 30 years. Owing to this, we are able to cure approximately 70–80 % children and adolescents with cancer, and in some types of cancer the survival rates are as high as 100 % provided that the diagnosis has been made early enough [4, 25]. The efficacy of cancer treatment depends on the degree of damage to the malignant cell population. That is why a single chemotherapy regimen may include several agents each of them having a different mechanism of action on tumour cells. As a result, they show a cumulative adverse effect on various organs and organ systems.

Being an important side effect of anthracyclines, cardiotoxicity may limit the efficacy of cancer therapies in the acute phase (i.e. during the treatment) and induce the long-term sequelae, observed years after treatment completion in childhood cancer survivors. Cardiovascular complications differ in type and severity depending on the actual cancer treatment. Cancer survivors tend to develop heart failure, ischaemic heart disease and cerebrovascular incidents more often than the general population. The cardiovascular mortality rates among childhood cancer survivors are ≤tenfold higher compared to the age-matched controls [35, 44]. Chemotherapy, radiation therapy and new biological therapies, used as a stand-alone treatment or in combination, constitute an important risk factor and predispose the patients to develop such complications.

## Objective

### Anthracycline Cardiotoxicity: Pathophysiology

Owing to their high efficacy, anthracycline antibiotics are included in numerous chemotherapeutic regimens used in treatment of solid tumours and blood malignancies, both in children and adults. Anthracyclines exert their main therapeutic effect by means of inserting (intercalation) between the base pairs in the DNA double helix and inhibiting the enzymatic activity of topoisomerase-II, DNA and RNA polymerases, helicases and DNA repair enzymes, which ultimately inhibits tumour cell proliferation [17].

The toxic effect of anthracyclines on cardiovascular system leads to the direct loss of cardiomyocytes, decreased cardiac muscle contractility and damage to the microvasculature. Furthermore, by affecting cardiac progenitor cells and fibroblasts, anthracyclines make it more difficult for the already-weakened heart to recover from injuries and the activity of other stressors, such as comorbidities or individual sensitivity [12].

Anthracycline cardiotoxicity involves multiple complex mechanisms, which have not been fully understood despite long-term research. The oxidative stress hypothesis is one of the commonly accepted cellular mechanisms to provide the potential explanation of cardiotoxicity. According to this theory, reactive oxygen species (ROS) and free radical formation directly trigger cardiac damage in cancer survivors. Anthracyclines may induce ROS formation via the enzymatic pathway, aglycone redox reaction and by forming iron complexes. The free radicals target DNA molecules and proteins, but also cellular membranes which contain large amounts of phospholipids. Free radicals damage DNA and cause lipid peroxidation, which in turn may lead to cell death and large-scale organ damage. The dynamics of the oxidative injury reflects and results from both oxidative stress and the decreased antioxidant levels. Cardiac muscle is particularly susceptible to free radicals generated by anthracycline antibiotics. Cardiomyocytes contain low levels of free radical scavengers, such as catalase and glutation peroxidase, which may cause their increased susceptibility to the damage by ROS [5, 21].

However, newer studies show that the free radical theory does not provide explanation to all aspects of anthracycline toxicity. Although the precise molecular and cellular cardiotoxicity mechanisms have not been fully explained, it is commonly thought to originate from cardiomyocyte apoptosis or necrosis and the sarcomere dysregulation and dysfunction [12]. Anthracyclines potentially damage several major structural proteins, which regulate cardiac muscle contractility. One of them is titin, the myofilament forming protein. Titin additionally regulates cardiac systolic function and sarcomere resting at diastole. Proteolysis and titin decomposition may therefore lead to systolic and diastolic dysfunction, typically present in patients treated with anthracyclines [11, 12]. Another major structural sarcomere protein, suggested as a potential target site of anthracyclines, is dystrophin, whose damage may increase the risk of dilated cardiomyopathy [10, 12].

However, there are also other mechanisms which contribute to the toxic effect of anthracyclines on the cardiac muscle. Anthracyclines may irreversibly disturb energy production in cardiomyocytes, interfering with their ability to induce the adequate contraction. They may also interfere with ATP production in cardiomyocytes, reduce the expression of mitochondrial RNA (mRNA) for sarco/endoplasmic reticulum Ca^2+^-ATPase (SERCA), decrease the activity of glutation peroxidase within the cardiac muscle and induce breathing difficulties by damage to mitochondrial DNA [12, 50].

Eventually, understanding the role of signalling proteins in cardiovascular system may help determine the actual anthracycline cardiotoxicity mechanisms, which will broaden our knowledge on the toxicity pathway itself and contribute to its earlier detection. Owing to the synergistic cardiotoxicity potential of anthracyclines and trastuzumab, monoclonal antibody which disrupts the ErbB2 signalling pathway, neuroregulin (NRG-1) has attracted increasing attention. Neuroregulin is a growth factor, endogenous ligand of the ErbB2 protein. Having been activated by binding to its ligand (i.e. neuroregulin-1) ErbB2 forms the ErbB4 dimer, thus activating the tyrosine kinase signalling pathway cascade, which promotes cardiomyocyte regeneration. After anthracycline administration, the level of neuroregulin decreases, which may suggest the potential toxicity mechanism [11, 17]. Recent studies show that heart failure observed in adult breast cancer survivors treated with anthracyclines and trastuzumab, can be attributed to cardiomyocyte apoptosis and necrosis, triggered by the direct anthracycline activity and trastuzumab-induced inhibition of cardiomyocyte regeneration pathway [11, 14]. The synergistic effect of trastuzumab and anthracyclines indicates the importance of crosstalk between the significant cardiomyocyte regulation pathways and sarcomere pathways, which are typically linked to the anthracycline toxicity. Furthermore, these results confirm the hypothesis that the anthracycline-induced cardiomyocyte damage combined with inhibition of physiological cardiomyocyte regeneration pathway by other stressors increases the risk of heart failure or other clinical manifestations of cardiotoxicity [12].

### Clinical Manifestation of Anthracycline Cardiotoxicity

The effect of anthracycline-based chemotherapy on human heart may be manifested differently in different individuals. The most typical clinical manifestations of cardiac muscle damage are as follows: asymptomatic ECG abnormalities, mild blood hypotension, cardiac arrhythmias, electrical conduction dysfunction, myocarditis, pericarditis, acute myocardial infarction, heart failure, and chronic dilated and/or restrictive cardiomyopathy [1, 5, 21, 33, 45].

That toxic effect of anthracyclines on cardiac muscle can manifest at any stage of treatment. Based on the time of onset, the following classification has been proposed:

- *Acute or subacute cardiotoxicity*—which develops during anthracycline-based chemotherapy, after a single administration or several cycles with a low cumulative dose, or within a year from treatment completion. This type of injury does not imply cardiomyocyte loss, so it is typically transient, reversible and non-dose dependant. It affects less than 1 % of patients. This form of cardiotoxicity is manifested clinically by paroxysmal arrhythmia, pericardial exudate, left ventricular dysfunction and—episodically—sudden cardiac death [21, 31].
- *Early*-*onset chronic cardiotoxicity*—which develops in less than 1 year following completion of cancer treatment and may be progressive. It affects approximately 2 % of patients [1, 5, 21, 31, 45].
- *Chronic progressive late*-*onset cardiotoxicity*—which is dose dependant, develops within more than 1 year, and often many years after cancer treatment completion. It is related to the irreversible cardiomyocyte loss. In most cases, it remains asymptomatic for many years as the subclinical anthracycline-induced cardiomyopathy. It is the most commonly reported cardiac muscle injury, which may affect—according to different authors—5 up to 57 % of cancer patients [30]. However, with time, it may lead to the irreversible heart injury which is symptomatized by the congestive heart failure or myocardial infarction [1, 5, 21, 45].

The recent research shows that the asymptomatic diastolic dysfunction, which is a common feature observed in many cancer survivors, is the earliest noticeable cardiac abnormality [20, 37]. By increasing calcium ion levels at diastole, anthracyclines may increase the left ventricular wall stiffness, simultaneously elevating the interstitial fluid pressure. This decreases the coronary arterial compliance which may lead to cardiac ischaemia. Subsequently, the abnormalities of left ventricular geometry develop (ectasia) followed by contractile dysfunction [36, 37]. Therefore, the left ventricular diastolic dysfunction bridges a gap between cancer treatment and cardiac ischaemia and/or systolic dysfunction. Diastolic dysfunction and the resulting decreased coronary arterial compliance make the heart more susceptible to comorbidities, which interfere with the coronary blood flow or increase the oxygen demand, such as early atherosclerosis or blood hypertension. The diastolic dysfunction may progress to systolic failure in such mechanisms as inhibition of or other stressors to human endothelial receptor-2 (HER-2), which is crucial for myocyte survival and normal myocardial contractile function [37].

### Risk Factors of Anthracycline Cardiotoxicity

Anthracycline toxicity and its clinical expression have multiple modifiers. Some of them can be linked to cancer treatment, whereas others are patient-related risk factors. There have been many studies to assess the risk of early and late anthracycline cardiotoxicity. Although their results have not been fully uniform, they helped determine the following risk factors for cardiovascular abnormalities in cancer survivors.

- *Total cumulative dose of anthracyclines*—the therapeutic efficacy of anthracyclines increases with the dose. However, so does their cardiotoxicity. In clinical studies on adults treated with doxorubicin, the risk for heart failure was 3–5 % with the cumulative dose of 400 mg m^2^, went up to 7–26 % with the dose below 550 mg/m^2^ and further increased up to 48 % with the dose of 700 mg/m^2^ [46]. Studies on childhood cancer survivors showed that the risk of obvious heart failure increases 11-fold in patient receiving doses over 300 mg/m^2^, as compared to ones treated with doses below 300 mg/m^2^ [29]. Nevertheless, severe cardiotoxicity symptoms were observed in patients receiving much lower doses of cytostatic agents, suggesting an important role of individual susceptibility to anthracyclines.
- *Time from cancer diagnosis and the moment of anthracycline treatment commencement*—the long-term studies in cancer survivors show that the risk of cardiovascular complications increases with the length of the follow-up [33].
- *Very young or advanced age of patient at the moment of diagnosis*, i.e. age below 4 years is associated with the increased risk of cardiotoxicity [33], although not all researchers confirm this association [30, 41].
- *Sex*—the potential association of anthracycline cardiotoxicity with female sex is emphasized. The actual mechanism has not been fully understood. However, sex-based differences in pharmacokinetics and pharmacodynamics of cytostatic drugs appear to play the role [33].
- *Black ethnicity* [31].
- *Down Syndrome* [31].
- *Chemotherapy combined with radiation therapy to the mediastinum* poses the highest risk of cardiac complications due to anatomical heart location [1, 21]. Early radiation-induced injury primarily involves the pericardium and manifests as acute pericarditis, which usually develops in several weeks following treatment completion. Coronary artery disease is the most common manifestation of late cardiovascular complications of radiation therapy with a typical late onset, i.e. in 10–15 years following radiation therapy. It was shown that ion radiation may induce or significantly accelerate atherosclerosis, both in patients with insignificant medical history and individuals with known cardiovascular risk factors. If myocardial damage occurs, restrictive cardiomyopathy will follow presenting as diastolic dysfunction. Additionally, the systolic heart function may become impaired and valvular heart disease, cardiac arrhythmias or conductivity disorder can develop [2, 6, 9].
- *Chemotherapy combined with the administration of other cardiotoxic agents* increases toxic effect of anthracyclines on cardiovascular system. The cardiotoxic effect of cyclophosphamide, iphosphamide, fluorouracil, bleomycin, vincristine, mitoxantrone and trastuzumab [5, 21] has been proven.
- *Cardiotoxicity may be induced or increased by the risk factors for cardiovascular diseases and the comorbidities*. They increase in number as the patient ages after the treatment is completed. These factors include blood hypertension, obesity and overweight, diabetes, dyslipidemia and imbalanced lifestyle with reduced physical exercise and/or smoking, alcohol misuse, stress and unhealthy diet. Similarly, other cardiovascular diseases, renal diseases, additional cardiovascular strain due to pregnancy, surgery or increased body weight can trigger cardiovascular symptoms in cancer survivors after a long-term asymptomatic period [5, 17, 28, 36, 44].

### Current Views on Cardiotoxicity

- The lifelong risk of cardiotoxicity

Understanding molecular assumptions of anthracycline-associated cardiotoxicity as well as knowledge and the detailed analysis of risk factors has changed the cardiotoxicity profile of this drug family. As the anthracycline doses were reduced, the incidence of acute cardiotoxicity plummeted, but the incidence of late cardiovascular complications did not, which may suggest that there is no anthracycline dose to be completely safe for human heart. The presence of a long-term, lifelong anthracycline cardiotoxicity risk has not been explained yet. It is known, however, that anthracyclines are eliminated from the myocardium quite fast and their level quickly drops below the acute toxicity threshold [37, 42]. It was initially attributed to mitochondrial dysfunction caused by exposure to doxorubicin, which led to the assumption that these abnormalities persist despite lack of continuous exposure to doxorubicin [32, 37]. However, the preliminary results from the animal models were not confirmed by studies on humans. It is thought currently that doxorubicin and daunomycin conversion to highly toxic C-13 alcohol metabolites such as doxorubicinol and daunorubicinol offers a better explanation of the lifelong risk of anthracycline cardiotoxicity. The said anthracyclines are poorly eliminated from cardiomyocytes and accumulate therein, which is a long-term sequelae of their presence within the cardiac muscle. Thus, it seems that alcohol metabolites of anthracycline antibiotics, which are even more toxic than their original compounds, may induce both early-onset symptoms (i.e. occurring during the chemotherapy) and late-onset symptoms (i.e. occurring many years after cancer treatment completion) [37]. Further research is required in order to address this issue.

- Individual variability

The non-invasive tests showed that 50 % of patients treated with anthracyclines in childhood (study group) had some asymptomatic myocardial dysfunction, which manifested as symptomatic incidents only in 5 % [39]. These data are similar in the group of adult cancer survivors, as well [23]. Individual and interracial variability may explain why different patients receiving similar treatment develop different symptoms of cardiotoxicity. It is currently assumed that the presence of gene polymorphisms which modify the pharmacodynamic properties of anthracyclines may result in variable cardiotoxicity effect in different individuals [15, 49].

Recent studies confirm the role of functional polymorphisms of genes coding for carbonyl reductases (CBR1 and CBR3), which regulate anthracycline conversion to their alcohol metabolites, as the primary mechanism in the etiopathogenesis of chronic anthracycline cardiomyopathy in patients treated with daunorubicin. Homozygosity for the major allele (G) in the CBR3 gene seems to increase the risk of anthracycline-associated cardiomyopathy at the exposure to low-to-moderate doses [7, 8]. The clinical importance of these results is being verified.

- Multiple hit hypothesis

The risk of cardiotoxicity in cancer survivors increases significantly at the presence of concomitant risk factors for cardiovascular diseases, such as blood hypertension, hyperlipidaemia or imbalanced lifestyle [36]. The available evidence also suggests that compared to age-matched healthy individuals, cancer survivors—initially with insignificant medical history—were diagnosed with the higher number of comorbidities and their level of physical activity dropped [13, 26]. This indicates that potentially reversible asymptomatic cardiotoxicity associated with the ‘safe doses’ of anthracyclines or other chemotherapeutic agents may progress to symptomatic incidents with overlapping risk factors which increase in number after the treatment is completed [37]. This is the so-called multiple hit hypothesis, which explains the late-onset cardiotoxicity as being the cumulative result of multiple drug-induced and non-drug-induced injuries to the cardiac muscle [26, 37]. That is why the direct effect of cytostatic drugs and imbalanced lifestyle decrease the cardiovascular reserve, increasing the risk of cardiovascular diseases and the premature cardiovascular death.

### Diagnosis and Monitoring of Cardiac Abnormalities

Standard management during anthracycline-based chemotherapy involves cardiac function assessment prior to treatment, monitoring potential cardiotoxicity during the therapy as well as a long-term follow-up after the chemotherapy is completed. In order to evaluate myocardial abnormalities laboratory tests, genetic tests and cardiovascular diagnostic imaging have proved to be useful.

Such biochemistry markers of necrosis as T-troponin, I-troponin and CKMB are useful in the acute phase of myocardial injury. They are elevated very early, often immediately after the first administration of anthracyclines. That is why they do not make good diagnostic markers of chronic cardiac injury. Similarly, more research on larger patient samples is needed in order to confirm the applicability of natriuretic peptide assays (NT-pro ANP and NT-pro BNP) in detecting late anthracycline cardiotoxicity [17]. Levels of these oligopeptide neurohormones correlate with the left ventricular ejection fraction (LVEF) and pulmonary capillary wedge pressure (PCWP). They do not meet the screening test criteria; however, their concentration within the reference ranges excludes heart failure in over 90 % of cases [40]. The search for new cardiotoxicity markers has also been continued. The following substances are proposed as potential markers: endothelin-1 (ET-1), fatty-acid-binding protein (FABP), cytokines and intercellular adhesion molecule-1 (ICAM-1) being the markers of endothelial damage as well as tissue plasminogen activator (TPA) and plasminogen activator inhibitor-1 (PAI-1) being the components of plasma fibrinolytic system [3]. Another approach proposes genetic testing aiming at early identification of patients at high risk of cardiotoxicity. Modification of cancer treatment in these patients may help avoid severe cardiovascular complications.

The next stage of diagnosis involves diagnostic imaging and LVEF assessment. Echocardiography is the most commonly available and used cardiovascular diagnostic imaging technique. It facilitates precise evaluation of heart and large vessel morphology as well as diagnosis of systolic and diastolic dysfunction. Three-dimensional echocardiography and quantitative assessment of intrinsic regional myocardial deformation (strain and strain rate technique) as well as tissue Doppler technique are the imaging techniques hoped to improve the precision and reproducibility of echocardiographic cardiac function parameters. The key role of echocardiography in the diagnosis of chemotherapy-induced cardiotoxic damage is primarily due to the variability of assessed parameters, the non-invasive nature of the procedure and the possibility to repeat it as often as necessary [24, 27].

Radionuclide angiography (RNA) is an alternative method for the diagnosis of cardiotoxic damage. Scintigraphy is also used for heart imaging in oncology, as it enables the assessment of left ventricular function. This includes such techniques as multiple-gated acquisition scintigraphy (MUGA) with the use of technetium-99m administered intravenously as a contrast agent, or indium-111 antimyosin antibody scintigraphy. Metabolic dysfunction, fibrosis and necrosis can be evaluated using the single-photon emission computed tomography (SPECT) and positron emission tomography (PET). Cardiac muscle biopsy is the most sensitive method to assess the cardiotoxicity at the cellular level. It is an invasive procedure, which limits its use to carefully selected cases. Similarly, other diagnostic techniques mentioned above should also be used after careful analysis of risk and cost against benefits, in selected patients with known indications. They are rarely used for screening or as a part of cancer treatment monitoring. They are not commonly used due to limited availability and high cost [16, 22, 38, 48].

### Anthracycline Cardiotoxicity Prevention

The main purpose of all preventive strategies is to minimize cardiotoxicity and to improve the efficacy of cancer treatments. The currently used anthracycline cardiotoxicity-preventive strategies include:

- *Limited cumulative anthracycline dose*—it is one of the most commonly accepted heart damage prevention methods. Therefore, the recommended highest doxorubicin dose should fall within the range of 400–550 mg/m^2^ and should not exceed 240 mg/m^2^ in most children and adolescents [17, 43, 44]. However, it should be emphasized that there is no completely safe dose of anthracyclines and even the lowest dose can induce severe cardiotoxicity.
- *Another subject for a discussion is the route of administration*, e.g. whether anthracyclines should be administered as a short quick bolus or a slow infusion. It is likely that heart injury does not depend on the cumulative dose only but also on peak serum anthracycline levels as well. Despite heterogeneous study results, it should be assumed that anthracycline administration as a long-term (over 48 h), continuous infusion can have the cardioprotective effect by means of decreasing the maximum serum drug concentration. Based on this assumption, many paediatric treatment protocols recommend continuous infusion, although long-term observation does not support its efficacy [17, 34].
- *The use of anthracycline analogues and liposomal anthracyclines*: Anthracycline analogues such as epirubicin, idarubicin or mitoxantrone are characterized by the weaker toxic effect on the cardiac muscle. Clinical studies showed lower cardiotoxicity of epirubicin, which does not accumulate within the cardiac muscle as doxorubicin does, whereas its efficacy is only slightly decreased. Currently, the so-called liposomal anthracyclines, also referred to as third-generation anthracyclines, are frequently used. The use of modern technology made it possible to transport the doxorubicin on a liposomal carrier and to prolong the time it remains within the circulation, just like the continuous infusion. Liposomes are capable of transporting the active substance selectively to the neoplastic tissue, which limits the anthracycline contact with healthy cells and tissues, thus limiting their toxicity to the cardiac muscle [18, 19]. The use of pegylated doxorubicin additionally decreases the risk of heart damage. However, liposomal anthracycline forms are very expensive, which limits their use.
- *Cardioprotective agents*—there have been attempts to use the known drug interactions in order to decrease anthracycline cardiotoxicity. The Q10 coenzyme, vitamin E and carnitine, which are known for their antioxidant properties, have been used. Another possibility is to use dexrazoxane (Cardioxane) which exerts its effect as iron chelator and inhibitor of free radical formation in the heart. The preliminary results of clinical trials of dexrazoxane on paediatric cancer patients indicate its cardioprotective efficacy at the early stages of treatment. The long-term effect, though, still has to be confirmed. It has been reported that this substance may reduce the efficacy of cancer therapies [47]. Therefore, further research is necessary.
- *It is particularly important to eliminate the classic* cardiovascular risk factors in cancer survivors. Comorbidity prevention and lifestyle modification can be used in these patients. Treatment can also be commenced earlier and more aggressively compared to the general population [17, 28, 34, 44].

## Summary and Conclusion

To sum up, it should be emphasized that the effective cancer treatment in children and adolescents is a huge success of modern medicine. As a result, the population of childhood cancer survivors is becoming more numerous each year. Considering the anticipated progression-free survival, the measures should be taken in order to limit the late effect of cancer treatment on other organs and systems, including cardiovascular system. That is why, having in mind the quality of life of cancer survivors, it is so important to understand the key mechanisms of toxic effect of anthracyclines on cardiovascular system, develop effective prevention and treatment strategies as well as cardiovascular monitoring system.
